# Impact of menopausal hormone therapy on influenza complications in women: a systematic assessment study

**DOI:** 10.1080/07853890.2025.2534095

**Published:** 2025-07-17

**Authors:** Yu-Hsiang Shih, Chiao-Yu Yang, Chia-Chi Lung

**Affiliations:** ^a^ Department of Obstetrics and Gynecology, Taichung Veterans General Hospital, Taichung, Taiwan; ^b^ Department of Public Health and Institute of Public Health, Chung Shan Medical University, Taichung, Taiwan; ^c^ Department of Occupational Health Nursing Center, Chung Shan Medical University Hospital, Taichung, Taiwan; ^d^ Department of Health Policy and Management, Chung Shan Medical University, Taichung, Taiwan; ^e^ Department of Family and Community Medicine, Chung Shan Medical University Hospital, Taichung, Taiwan

**Keywords:** Menopausal hormone therapy, influenza, complication

## Abstract

**Background:**

Research on the effects of Menopausal Hormone Therapy (MHT) on influenza outcomes has been limited.

**Methods:**

This retrospective cohort study utilized data from the TriNetX U.S. Collaborative Network between January 1, 2010, and December 31, 2019, targeting individuals aged 46 to 60 diagnosed with influenza who had not received the influenza vaccination in the prior year. Participants were divided into two cohorts: the MHT cohort, which included individuals who had used estrogen within the preceding 3 months, and the non-MHT cohort, consisting of those who had not used estrogen during the same period. Propensity score matching (PSM) was employed to balance key demographic and clinical variables, including age, race, hypertension, diabetes mellitus, dyslipidemia, pulmonary diseases, and heart diseases. The primary analysis was the assessment of respiratory morbidity within three months following the influenza diagnosis, while secondary analysis included the evaluation of patients with pre-existing lung or heart diseases and those who received antiviral medication.

**Results:**

After PSM, each cohort included 15,136 women. Women aged 46–60 who used MHT experienced a significant reduction in lung complications, with the risk of influenza pneumonia or respiratory failure decreasing by approximately 40%. However, among patients with chronic conditions such as diabetes, hypertension, hyperlipidemia, lung disease, or heart disease, MHT did not demonstrate a clear protective effect. Similarly, in patients who received antiviral treatment following influenza infection, the MHT group showed no significant reduction in the risk of respiratory complications.

**Conclusion:**

In conclusion, MHT was associated with a significant reduction in the risk of lung complications in women aged 46–60 without chronic conditions.

## Introduction

Influenza is a viral respiratory illness that poses a significant global health threat. Previous studies estimate that influenza is responsible for 291,243 to 645,832 deaths annually worldwide [1]. Vaccination has been shown to effectively reduce the risk of influenza-related complications [2]. In the United States, the average incidence of influenza among adults aged 50 to 64 over the past decade has been 11.6% [3]. Furthermore, according to a report by Yandrapalli et al. the hospitalization rate due to influenza was significantly higher in the 45–64 age group, reaching 36.8% in the United States [4]. However, according to 2023 Centers for Disease Control and Prevention survey data, only 41.2% of individuals aged 50–64 years reported that they ‘definitely will’ get the flu vaccine [5].This indicates that a substantial number of women in the menopausal age group remain at risk of severe influenza infection without the protection of vaccination.

Menopausal hormone therapy (MHT) has been the focus of extensive research, particularly regarding its potential effects on a range of health outcomes [6]. Estrogen, a key component of MHT, is known to exert protective effects primarily through its immunomodulatory and anti-inflammatory properties. It influences macrophage polarization by promoting the differentiation toward M2 macrophages, which play a critical role in controlling infectious diseases [7]. Supporting this, animal studies have demonstrated that mice treated with 17β-estradiol (E2) exhibited a shorter course of influenza infection, delayed symptom onset, and reduced overall morbidity and mortality [8]. Despite these findings, real-world epidemiological evidence on the impact of MHT in the context of influenza remains limited. To address this gap, we conducted a comprehensive analysis using the TriNetX database, a large-scale, representative network of electronic health records from multiple healthcare organizations. Our study aimed to assess the association between MHT use and influenza-related outcomes—including respiratory complications and all-cause mortality—among middle-aged women undergoing perimenopause or menopause.

## Method

### Data source

This retrospective study utilized electronic health record data from the TriNetX US Collaborative Network, a global federated platform that aggregates real-world data from over 120 healthcare organizations. TriNetX integrates diverse clinical information, including diagnoses, medications, laboratory tests, and procedures. Data standardization is achieved through syntactic and semantic harmonization. The TriNetX Common Data Model structurally aligns data from various Electronic Health Record systems and international standards (e.g. Observational Medical Outcomes Partnership, Fast Healthcare Interoperability Resources). Semantic harmonization maps diagnosis to ICD-9/10, medications to RxNorm, and lab tests to Logical Observation Identifiers Names and Codes to ensure consistency. Unmapped concepts are tracked and regularly updated.

While this study primarily used structured data, TriNetX also applies natural language processing to extract clinical data from unstructured notes. For missing data, TriNetX excludes records with missing key variables without imputation; thus, our analysis is based on a complete-case dataset. The missing rate for key variables is reported to be below 2%. All analyses followed the platform’s standardized processing protocols and quality control guidelines [9].

### Ethics statement

Ethical considerations were duly addressed, with an informed consent waiver granted owing to the anonymous nature of the data and compliance with regulatory guidelines such as the Health Insurance Portability & Accountability Act and the General Data Protection Regulation. Approval for this study was obtained from the Institutional Review Board committee of Taichung Veterans General Hospital. (CE24226C). The study was conducted in accordance with the principles of the Declaration of Helsinki.

### Cohort description

**Figure 1.**
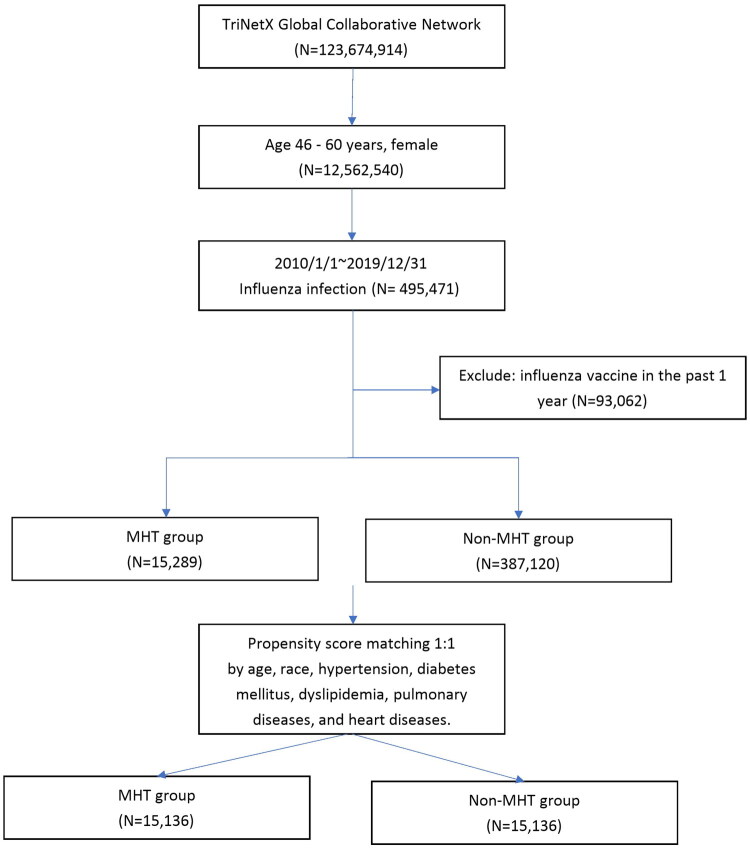
Flowchart of study cohort selection of women aged 46–60 years with influenza infection (2010–2019) from the TriNetX Global Collaborative Network, excluding those vaccinated in the past year, followed by 1:1 propensity score matching based on demographic and clinical characteristics.

The study period spanned from January 1, 2010, to December 31, 2019. The study population included individuals aged 46 to 60—women with menopausal symptoms or who had reached menopause—who were diagnosed with influenza and had not received an influenza vaccination within the preceding year. Participants were categorized into two groups based on hormone use: the MHT group comprised those who had used estrogen with or without progesterone within the three months prior to diagnosis, while the non-MHT group consisted of individuals who had not used estrogen and progesterone during the same period. The use of vaginal estrogens was excluded from this study, as systemic absorption from vaginal estrogen has been shown to be minimal, and thus its impact on the immune system is likely limited [10]. To ensure comparability between the two groups, propensity score matching (PSM) was employed to balance key demographic and clinical variables. These included age, race, hypertension, diabetes mellitus, disorders of lipoprotein metabolism, asthma, bronchiectasis, interstitial lung disease, chronic obstructive pulmonary disease (COPD), heart failure, cardiomyopathy, ischemic heart disease, and cerebrovascular disease. Figure 1 illustrates the study flowchart.

Baseline data were collected at the index date, which was determined based on the diagnosis of influenza. The primary analysis was the assessment of respiratory morbidity within three months following an influenza diagnosis, while the secondary analysis included evaluating patients with pre-existing lung or heart disease, those with comorbidities such as hypertension, diabetes mellitus, or hyperlipidemia, and patients who received antiviral medication (Peramivir, Oseltamivir, Amantadine, Baloxavir marboxil, Zanamivir). The drugs and diseases mentioned in this article with their corresponding codes are provided in Supplementary Table S1.

### Statistical analysis

This study utilized the TriNetX platform to perform PSM to establish 1:1 matched groups with similar baseline characteristics. The matching procedure employed a greedy nearest neighbor algorithm with a caliper width of 0.1 times the pooled standard deviation of the logit of the propensity score, in accordance with standard practice. Balance between matched groups was evaluated using the Standardized Mean Difference (SMD), with values less than 0.1 considered indicative of sufficient balance. Stratified analyses employed independent matching based on relevant covariates. Following matching, hazard ratios (HRs) and 95% confidence intervals (CIs) were calculated using chi-square tests. Cumulative incidence was assessed by Kaplan–Meier curves and log-rank tests. Statistical significance was set at a two-sided p-value < 0.05. All analyses were conducted using the TriNetX online platform.

### Result

A total of 402,409 patients were involved in the study, with MHT cohort including 15,289 patients and non-MHT cohort comprising 387,120 patients. PSM was utilized to ensure a balanced comparison between the two cohorts. Following matching, both the MHT cohort and non-MHT cohort comprised 15,136 patients each. The average age at the index was 52.9 years old, with a diverse representation of ethnicity: 74.7% White, 9.1% Black or African American, and 3.7% Asian. There were no discernible differences in the prevalence of hypertension, diabetes mellitus, disorders of lipoprotein metabolism, asthma, respiratory failure, interstitial pulmonary disease, chronic obstructive pulmonary disease (COPD), heart failure, cardiomyopathy, ischemic heart disease and cerebrovascular disease between the two patient cohorts (Table 1).

**Table 1.** Baseline characteristics of study population.

	Before PSM			After PSM		
	MHT	Non-MHT	SMD	MHT	Non-MHT	SMD
	(*n* = 15,289)	(*n* = 387,120)		(*n* = 15,136)	(*n* = 15,136)	
Age (mean ± SD)	52.9 ± 4.5	53.0 ± 4.4	0.018	52.9 ± 4.5	52.9 ± 4.5	0.002
Race, n(%)						
White	11,302(74.7)	229,765(60.0)	0.317	11,300(74.7)	11,306(74.7)	0.001
Black or African American	1,379(9.1)	69,021(18.0)	0.262	1,379(9.1)	1,379(9.1)	0.001
Asian	553(3.7)	22,171(5.8)	0.101	553(3.7)	549(3.6)	0.001
Comorbidities, n(%)						
Hypertension	2,642(17.5)	62,372(16.3)	0.031	2,641(17.4)	2,648(17.5)	0.001
Diabetes mellitus	1,029(6.8)	28,929(7.6)	0.029	1,029(6.8)	1,012(6.7)	0.004
Disorders of lipoprotein metabolism and other lipidemias	2,486(16.4)	42,613(11.1)	0.154	2,484(16.4)	2,513(16.6)	0.005
Asthma	2,024(13.4)	33,795(8.8)	0.145	2,022(13.4)	1,998(13.2)	0.005
Bronchiectasis	222(1.5)	6,497(1.7)	0.018	221(1.5)	203(1.3)	0.01
Interstitial pulmonary diseases	129(0.9)	1,970(0.5)	0.041	127(0.8)	90(0.6)	0.029
chronic obstructive pulmonary disease	279(1.8)	8,461(2.2)	0.026	279(1.8)	252(1.7)	0.014
Heart failure	182(1.2)	6,731(1.8)	0.046	182(1.2)	182(1.2)	0.001
Cardiomyopathy	127(0.8)	3,157(0.8)	0.002	127(0.8)	120(0.8)	0.005
Ischemic heart disease	328(2.2)	9,631(2.5)	0.023	328(2.2)	311(2.1)	0.008
Cerebrovascular diseases	278(1.8)	7,779(2.0)	0.014	278(1.8)	271(1.8)	0.005

PSM: propensity score matching. SMD: standardized mean difference. MHT: menopausal hormone therapy.

Our study revealed a significant reduction in influenza-related health issues—including pneumonia, respiratory failure, acute respiratory distress syndrome (ARDS), intubation, and shock—among MHT users. The hazard ratio (HR) for influenza-related pneumonia in the MHT group was 0.62 (95% CI: 0.54–0.70), indicating a markedly reduced risk. Similarly, the HR for respiratory failure was 0.64 (95% CI: 0.50–0.82), demonstrating MHT’s protective effect. While the risks for ARDS and intubation appeared reduced, the results did not reach statistical significance. The HR for ARDS was 0.49 (95% CI: 0.22–1.09), and for intubation, the HR was 0.69 (95% CI: 0.38–1.23). Additionally, the risk of shock was significantly reduced, with an HR of 0.53 (95% CI: 0.30–0.95). Regarding mortality, the risk was lower in the MHT group, but this reduction did not reach statistical significance (HR: 0.78, 95% CI: 0.53–1.14) (Table 2).

**Table 2.** HRs and 95% CIs for the risk of influenza-associated morbidity stratified by the status of MHT use (*n* = 15,136).

	Patients with outcome (n)		
	MHT	Non-MHT	HR (95% CI)
Respiratory system			
Influenza pneumonia	351	554	0.62(0.54,0.70)
ARDS	10	18	0.49(0.22,1.09)
Respiratory failure	100	153	0.64(0.50, 0.82)
Intubation	19	27	0.69(0.38,1.23)
Systemic			
Shock	18	33	0.53(0.30,0.95)
Deceased	48	60	0.78(0.53,1.14)

MHT: menopausal hormone therapy. ARDS: acute respiratory distress syndrome.

In individuals with hypertension, diabetes mellitus, or hyperlipidemia who were infected with influenza, MHT did not confer significant benefits for respiratory outcomes, shock, or mortality. Similarly, among patients with pre-existing lung or heart disease, MHT showed no meaningful improvement in these outcomes. Across both subgroups, no statistically significant differences were observed in the incidence of respiratory complications and shock. Specifically, in patients with lung diseases—such as COPD, asthma, pulmonary fibrosis, or lung cancer—MHT did not demonstrate protective effects against influenza-related pneumonia, ARDS, respiratory failure, or the need for intubation. Likewise, among those with heart conditions, including heart failure, acute myocardial infarction, and ischemic cardiomyopathy, MHT did not reduce the risk of pulmonary complications. Notably, an increased risk of mortality was observed in heart disease patients using MHT (HR 2.62; 95% CI: 1.10–6.28) (Table 3).

**Table 3.** Number of cases and hazard ratios (HRs with 95% confidence intervals) for influenza-associated morbidity, stratified by MHT use in patients with chronic diseases.

	Patients with diabetes or hypertension or hyperlipidemia (*n* = 1,706)			Patients with lung disease (*n* = 1,998)			Patients with heart disease (*n* = 208)		
	MHT	Non-MHT	HR (95% CI)	MHT	Non-MHT	HR (95% CI)	MHT	Non-MHT	HR (95% CI)
Respiratory system									
Influenza pneumonia	119	125	0.94(0.73,1.21)	121	103	1.17(0.90,1.52)	22	23	0.96(0.53,1.72)
ARDS	10	10	1.99(0.36,10.85)	10	10	0.99(0.25,3.95)	10	10	1.00(0.06,15.96)
Respiratory failure	55	46	1.19(0.80,1.76)	54	51	1.05(0.72,1.54)	29	21	1.40(0.80,2.45)
Intubation	12	10	1.49(0.61,3.64)	10	10	1.32(0.46,3.81)	10	10	4.58(0.99,21.18)
Systemic									
Shock	10	10	0.88(0.34,2.28)	10	10	0.98(0.35,2.81)	10	10	2.05(0.62,6.84)
Deceased	28	18	1.54(0.85,2.78)	15	21	0.70(0.36,1.37)	18	10	2.62(1.10,6.28)

Numbers in the table represent the number of patients with each outcome in the MHT and non-MHT groups within each chronic disease category, followed by the corresponding hazard ratios (HRs) with 95% confidence intervals (CIs).

MHT: menopausal hormone therapy. ARDS: acute respiratory distress syndrome.

Chronic diseases are primarily categorized as patients with diabetes or hypertension or hyperlipidemia (*n* = 1706), patients with lung disease (*n* = 1998), patients with heart disease (*n* = 208).

Upon further analysis, we observed that among patients who received antiviral treatment after influenza infection, the MHT group showed a reduced risk of influenza pneumonia and respiratory complications. However, similar to ARDS, intubation, shock, and mortality, these reductions did not reach statistical significance (Table 4).

**Table 4.** HRs and 95% CIs for the risk of influenza-associated morbidity stratified by antiviral medication use (*n* = 1,401).

	Patients with outcome (n)		
	MHT	Non-MHT	HR (95% CI)
Respiratory system			
Influenza pneumonia	151	179	0.81(0.65,1.00)
ARDS	10	10	0.96(0.14,6.81)
Respiratory failure	20	30	0.65(0.37,1.14)
Intubation	12	10	1.49(0.61,3.64)
Systemic			
Shock	10	10	0.65(0.18,2.29)
Deceased	10	10	0.48(0.15,1.61)

MHT: menopausal hormone therapy. ARDS: acute respiratory distress syndrome.

## Discussion

In our study, we found that women aged 46–60 who use MHT experienced a significant reduction in lung complications, with the risk of influenza pneumonia or respiratory failure decreasing by approximately 40%. However, among patients with chronic conditions such as diabetes, hypertension, hyperlipidemia, lung disease, or heart disease, MHT did not demonstrate a clear protective effect. Similarly, in patients who received antiviral treatment after influenza infection, the MHT group showed no significant reduction in the risk of respiratory complications.

Endogenous sex steroid hormones, such as 17β-estradiol (E2), possess immunomodulatory properties and regulate cellular immune responses during infection [11]. Ahmet Yalcinkaya et al. also noted that estrogen enhances immune responses, which may be beneficial in the context of infections [12]. Previous research has highlighted the antiviral properties of E2 and estrogenic chemicals against a range of viruses like HIV, HCV, Ebola virus, and HCMV [13–15]. However, it’s still unclear whether these effects stem from shared molecular mechanisms across different virus families or from E2’s broad impact on cellular gene expression pathways. In the context of influenza virus, Jackye Peretz et al. discovered that E2 and specific estrogenic compounds exhibit antiviral effects against influenza A virus infection [16]. These compounds were observed to reduce the peak viral titer, which coincided with a decrease in metabolic processes [16]. Supporting evidence from animal studies shows that activation of estrogen receptor-1 in female mice reduces pulmonary levels of proinflammatory cytokines and chemokines, leading to improved outcomes following influenza infection [17]. Furthermore, there is increasing evidence suggesting that hormonal changes during the menopausal transition could influence pulmonary function, lung vasculature, and respiratory diseases [18]. While the exact mechanisms through which the decline in estrogen affects respiratory health remains unclear, it is hypothesized that sex hormones may directly influence airway and immune cells, thereby impacting pathways relevant to respiratory diseases [18].

Influenza can indeed result in various lung complications, such as pneumonia and acute respiratory distress syndrome [19]. Influenza infections typically start in the upper respiratory tract, where epithelial cells become infected, triggering mucous production and activating the innate immune response [18]. Viral‐damaged airway epithelial cells attract immune effector cells like neutrophils, monocytes, and macrophages, which exacerbate inflammation and cause local tissue damage. Subsequently, the virus spreads to the alveoli, leading to viral pneumonia. The combined effects of epithelial cell death, hyper‐inflammation, and heightened airway permeability culminate in acute lung injury, often necessitating hospitalization [20]. In various inflammatory models of the lung and heart, estrogen has consistently demonstrated an anti-inflammatory effect, particularly at levels corresponding to proestrus to pregnancy [11]. In our study, we observed that the use of estrogen reduced the risk of lung complications following influenza infection, which may be attributed to the anti-inflammatory effects of estrogen [11].

Patients with chronic heart or lung diseases are considered high-risk for developing complications from influenza [19]. In our study, we observed that individuals with lung or heart diseases may not derive the same degree of benefit from MHT in reducing complications. Previous studies have indicated that influenza can worsen pre-existing chronic conditions, such as COPD and asthma, further impairing respiratory function [21]. Among individuals with pre-existing lung diseases, severe complications triggered by influenza infection may offset the protective effects of estrogen. Therefore, MHT did not demonstrate significant protective benefits in patients with chronic lung diseases.

Similarly, in patients with heart diseases, our study showed that MHT did not reduce the incidence of pulmonary complications. Furthermore, we observed an increase in all-cause mortality among heart disease patients using MHT. Previous report have noted that in individuals with atherosclerotic vascular disease, the use of estrogen does not offer significant cardiovascular protection and is associated with increased mortality [22]. Additionally, other studies have reported a higher risk of acute cardiovascular events and mortality during influenza infection [21]. These factors may explain why MHT does not provide significant benefits for patients with heart diseases.

We also observed that patients with diabetes, hypertension, or hyperlipidemia did not derive significant benefits from MHT. One study utilizing data from the US National Health and Nutrition Examination Survey 2007–2010 found that adults with metabolic syndrome were significantly more likely to exhibit a restrictive pattern of lung function [23]. Emerging evidence suggests that diabetes and widely used antidiabetic medications may influence the mechanisms, development, and progression of various lung diseases [24]. The same applies to hypertension; previous studies have shown that the combination of high blood pressure and the use of antihypertensive medications has the most pronounced negative effect on lung function, being associated with a decline in both Forced Expiratory Volume in 1 s (FEV_1_) and Forced Vital Capacity (FVC) [25]. Therefore, in patients with diabetes, hypertension, or hyperlipidemia, it is speculated that their lung function may be poorer compared to the general population. This could explain, as seen in our prior analysis, why this group and patients with lung diseases did not derive significant benefits from MHT.

Most current guidelines advocate for the early administration of antiviral medications, especially in individuals susceptible to severe illness [26]. Three classes of antiviral drugs—M2 blockers, neuraminidase inhibitors, and polymerase acid inhibitors that inhibit viral replication—have gained approval in numerous countries for the treatment of influenza infections [27]. The accessibility of these medications has significantly shortened the duration of illness, eased symptoms, and made a remarkable contribution to curbing the transmission and outbreak of influenza [27]. For example, Oseltamivir is the most recommended and used agent in influenza treatment. A multicenter observational study demonstrated a 38% reduction in mortality among patients in whom treatment was initiated within 48 h of symptom onset [28]. In our study, we found that among influenza patients with a history of MHT use, the administration of antiviral medications reduced the incidence of influenza pneumonia and respiratory failure, but the results did not reach statistical significance. This suggests that if patients can promptly use antiviral medications after contracting influenza, the benefits of MHT for women may become less apparent.

While our study is comprehensive, it is important to recognize specific limitations. Firstly, we did not differentiate between the types of influenza involved. Other limitations include the timeframe for the onset of complications following influenza infection and the inability to calculate cause-specific mortality. Additionally, we lack detailed information on the type, dosage, duration, and route of estrogen therapy, as well as whether progesterone was used concurrently. Many of these limitations stem from the nature of secondary data collected *via* the TriNetX platform. Although TriNetX aggregates data from over 120 healthcare organizations across the U.S., clinical events such as hospitalizations or deaths occurring outside the network may not be captured, introducing potential information bias. Since the study pooled patients with menopause diagnosis and those with symptoms of menopause, the results should be interpreted with consideration of this limitation. Future research should explore these issues in more depth and investigate underlying mechanisms and residual confounding.

## Conclusion

Overall, our study suggests that MHT may offer protective effects against pulmonary complications in otherwise healthy individuals; however, it does not confer significant benefits in patients with chronic conditions, and its impact on mortality remains unclear. These findings carry important implications for clinical practice and public health strategies in the management of influenza and its associated complications.

## Supplementary Material

Supplementary Table S1.docx

## Data Availability

The data that support the findings of this study are available in TriNetX official web site at https://trinetx.com/. The data related to this study will be shared upon reasonable request made to the corresponding author of the manuscript.
