# Supplementary material for: Impact of menopausal hormone therapy on influenza complications in women: a systematic assessment study
Source: Ann Med. 2025 Jul 17;57(1):2534095. doi: 10.1080/07853890.2025.2534095 (PMC12278453; doi:10.1080/07853890.2025.2534095)
Supplement: Supplementary Table S1.docx [file IANN_A_2534095_SM1895.docx]

**Supplementary Table S1. Medical Codes of Cohort study**

| **Variable** | | **Code(s)** |
| --- | --- | --- |
| **Influenza** | | **UMLS: ICD-10-CM: J10, J11**  **NLM: 1657128, 1657134, 1657131, 1312375, 1312376**  **UMLS: 31859-2, 31864-2, 34487-9, 38381-0, 40982-1, 43874-7, 43895-2, 49521-8, 49524-2, 53251-5, 55465-9, 5862-8, 5866-9, 72356-9, 76078-5, 76080-1, 80382-5, 80383-3, 82166-0, 82170-2, 85478-6, 92141-1, 92142-9** |
| **Influenza Vaccine** | | **UMLS: 90630, 90653, 90654, 90655, 90656, 90657, 90658, 90660, 90661, 90662, 90672, 90673, 90674, 90682, 90685, 90686, 90687, 90688, 90694, 90756, 86198006, Z23**  **NLM: 805524, 805551, 857917, 857919, 857921, 864701, 1005909, 1005929, 1005931, 1006250, 1116738, 1303855, 1304122, 1427020, 1427022, 1541617** |
| **Menopausal and other perimenopausal disorders** | | **ICD-10-CM: N95** |
| **Asymptomatic menopausal state** | | **ICD-10-CM: Z78.0** |
| **Estrogens** | | **NLM: ATC: G03C, HS300** |
| **Vaginal Estrogen** | | **NLM:VA:GU500** |
| **Peramivir** | | **RxNorm 619693** |
| **Oseltamivir** | | **RxNorm 260101** |
| **Amantadine** | | **RxNorm 620** |
| **Baloxavir marboxil** | | **RxNorm 2099995** |
| **Zanamivir** | | **RxNorm 69722** |
| **Asthma** | | **ICD-10-CM: J45** |
| **Interstitial pulmonary disease** | | **ICD-10-CM: J84** |
| **Bronchiectasis** | | **ICD-10-CM: J47** |
| **Chronic obstructive pulmonary disease** | | **ICD-10-CM: J44** |
| **Hypertension** | | **ICD-10-CM: I10-I1A** |
| **Ischemic heart disease** | | **ICD-10-CM: I20-I25** |
| **Cardiomyopathy** | | **ICD-10-CM: I42** |
| **Diabetes mellitus** | | **ICD-10-CM: E08-E13** |
| **Cerebrovascular disease** | | **ICD-10-CM: I60-I69** |
| **Disorder of lipoprotein metabolism** | | **ICD-10-CM: E78** |
| **Outcome** | **Influenza pneumonia** | **ICD-10-CM: J09-J18** |
|  | **Acute respiratory distress syndrome** | **ICD-10-CM: J80** |
|  | **Respiratory failure** | **ICD-10-CM: I96** |
|  | **Intubation** | **UMLS: CPT:31500** |
|  | **Shock** | **ICD-10-CM: R65.21** |
